# Reinforcement of single-walled carbon nanotubes on polydimethylsiloxane membranes for CO_2_, O_2_, and N_2_ permeability/selectivity

**DOI:** 10.1007/s11356-023-26962-x

**Published:** 2023-04-26

**Authors:** Bassem Fareed Felemban, Sadia Sagar Iqbal, Ali Bahadar, Nazia Hossain, Abdul Jabbar

**Affiliations:** 1 Department of Mechanical Engineering, College of Engineering, Taif University, P.O. Box 11099, Taif, 21955 Saudi Arabia; 2 Department of Physics, Lahore Garrison University, Lahore, Pakistan; 3 Department of Chemical and Materials Engineering, King Abdulaziz University, Rabigh, 21911 Saudi Arabia; 4 School of Engineering, RMIT University, VIC 3001 Melbourne, Australia; 5 Institute of Polymer and Textile Engineering and Technology, University of the Punjab, Lahore, Pakistan

**Keywords:** Single-walled carbon nanotubes, Modified membrane, Polydimethylsiloxane, Gas separation, Thermal properties, Mechanical properties

## Abstract

In this study, PDMS incorporated with SWCNTs have been fabricated via solution casting method for industrial applications and characterized by the analyses of SEM, FTIR, TGA, AFM, and MST. The modified membranes were further analyzed for CO_2_, O_2_, and N_2_ gas permeability. The strategic membranes have five different weight ratios (0.013, 0.025, 0.038, 0.050, 0.063) compared to neat PDMS membranes. The even distribution of SWCNTs in PDMS provided results that showed improvement in thermal stability. However, mechanical strength has been weakened with increased concentration of nanofiller because of the increase in the number of SWCNTs by increases that imperfections become more severe. The designed polymeric membranes with good thermal stability and adequate mechanical strength can be used for the selectivity and permeability of CO_2_, O_2_, and N_2_ gases. The effect of the PDMS-SWCNTs on gas permeability has been studied. 0.063 wt.% SWCNTs presented the maximum permeability of CO_2_ gas while maximum O_2_ and N_2_ gas permeability have been obtained by 0.013 wt.% SWCNTs. The ideal selectivity of mixed (50:50) gas conditions has been tested. The maximum CO_2_/N_2_ ideal selectivity was obtained by 0.050 and 0.063 wt.% SWCNTs while maximum O_2_/N_2_ ideal selectivity obtained by 0.050 wt.% SWCNTs. Therefore, the fabrication of this novel SWCNTs-PDMS membrane may lead to separating the industrial exhaust and be used as a potential membrane for environmental remediation in the future.

## Introduction

In the gas separation process, membrane technology plays a very influential role. A wide range of polymeric membranes is used in many industrial and commercial applications due to their excellent features. These polymeric gas separation membranes are very compact in size, have less capital cost, and have better modular configuration, leading to less power consumption and decreasing production costs (Quan et al. 2017). The carbon emission is very low from biogas compared to coal heating and liquefied petroleum (Hossain and Morni 2020). The environmental impact and energy consumption cost are significant challenges due to the increment in energy demand and mitigation of global climate warming. Many commercial and industrial applications use polymeric membranes to overcome these climate change impacts (Mubashir et al. 2021; Viannie et al. 2021).

Polymeric membranes have been applied in environmental applications like water purification and pre and post gas combustion due to their significant features. These membranes are also used in drug delivery devices and the separation of various gas mixtures. Polymeric membranes have great importance in CO_2_ separation from the air due to their energy-saving cost and operational process simplicity, which have become an excellent curiosity for researchers (Mubashir et al. 2018). The polymeric membranes have weak interactions between macromolecules, or the existence of a statistically distributed free volume, allowing gasses to permeate through the polymer. Hybrid polymeric membranes have increased permeability due to the increased contribution of diffusion components (Baker 2000; Iqbal et al. 2021; Viannie et al. 2021).

One of the most promising polymers is polydimethylsiloxane (PDMS). It has good chemical and thermal stability, biocompatibility, ease of use, chemical inertia, hyperplastic characteristics, and gas permeability at a low cost. Thus, PDMS has been used in several fields and systems (microfluidics/nanofluidics, electronic components, membranes for filtering and pervaporation, sensors, thermal devices, coatings, and others). With compromised mechanical properties, the tailored PDMS composites with nano-reinforcements can improve the properties of PDMS. The PDMS polymeric composite membranes are used in many gas separation applications and have significant importance due to their high permeability and flexibility in nature. Different inorganic, polymeric, and hybrid membrane materials are developed, and their challenges are studied (Javaid 2005; Pan et al. 2022). The block copolymer is synthesized by atom transfer radical polymerization technique for thermodynamic study. The cylindrical micelles of copolymers were confirmed by Flory–Huggins, and solubility parameters along the phase-separated X-ray analyze morphology. The fibrillar morphology of the blend membrane had good permeation and selectivity (Fishlock et al. 2018, Semsarzadeh and Ghahramani 2015, Shahapurkar et al. 2021). A freely suspended PDMS membrane is synthesized in situ. The amphiphilic properties of PDMS precursors make them suitable for water/air treatment (Bilotkach and Lee 2008). Spectral and thermal studies of PDMS with increasing concentrations of cross-linkers and nano-reinforcements were used to characterize gas selectivity applications (Alvaro and Roy 2005, Jadhav et al. 2020; Kammermeyer 1957). These membranes are advantageous for separation because of their permeability, low cost, good mechanical stability, ease of processability, and reasonably good selectivity (Adrees et al. 2019). The earlier study re-examined the relationship between CO_2_/CH_4_ selectivity and CO_2_ permeability. This study reported a modest shift in polymer-bound positions for gas pairs since 1991 (Jadhav et al. 2020; Robeson 2008).

The polymeric membranes have significantly less energy and are separated at a molecular level. It is observed that the separation properties of the membrane have improved with the addition of multi-walled carbon nanotubes (MWCNTs) (Mazari et al. 2021; Sanip et al. 2011). MWCNTs/PDMS composites were synthesized as membranes to evaluate their gas separation properties for the separation of H_2_ from CH_4_ gas species (Nour et al. 2013, Rutnakornpituk and Ngamdee 2006). PDMS membranes with varying concentrations of multi-walled carbon nanotubes were prepared to evaluate their gas separation performances of CO_2_, CH_4_, N_2_, and O_2_ (Berean et al. 2014; Mubashir et al. 2016; Silva et al. 2017). The evaluation of membranes using ZIF-62 metal–organic framework nano-hybrid dots was discussed for environmental remediation (Mubashir et al. 2022). The molecular-scale fabrication technique is used to fabricate unique CNT-ZIF-8-PDMS composite membranes with enhanced gas separation performance, mainly focusing on CO_2_ capturing (CO_2_/N_2_, CO_2_/H_2_) (Ashtiani et al. 2021; Reijerkerk et al. 2010). In the inert pyrolysis process, the polyimide siloxane was converted into the carbon-rich phase of the imide domains. Later, molecular sieving capabilities were checked for minor gasses O_2_/N_2_, CO_2_/N_2_, H_2_/N_2_, and He/N_2_. In polyimide siloxane, the siloxane domains are further converted into the silica-rich phase to enhance gas pathways (Gao et al. 2022; Park et al. 2004).

The effects of the SWCNTs upon exposure to high-energy protons, the stopping ranges for PDMS/SWCNT, and pure PDMS were simulated, and the results were compared to those obtained from the experiments (Li et al. 2013). An earlier experimental study (Kim et al. 2006) discussed the nanocomposite membranes consisting of single-walled carbon nanotubes embedded in a poly(imide siloxane) copolymer, evaluated their transport properties, and suggested that the single-walled carbon nanotubes (SWCNTs) offer an attractive additive for universally enhancing the gas permeability of polymers. The promising features of SWCNT/PIS membranes for augmentation of gas permeability urge us to study the processes for further development of gas permeability/selectivity. Therefore, this study emphasized an experimental attempt to determine the influence of SWCNTs for stimulating the properties of PDMS to explore the possibility of a higher efficient membrane compared to existing literature. Hence, the novelty of this study lies in exploring SWCNTs on PDMS membranes for CO_2_, O_2_, and N_2_ gas selectivity and permeability that may lead to the high potential of this modified membrane in industrial applications to separate exhaust waste more effectively in future research.

The main objective of this experimental study is to investigate the efficiency of gas separation and permeability and purify the polluted gas from industries which may lead to protecting humans and the ecosystem from harmful and toxic elements in exhausted gasses. Novel polymeric membranes of SWCNTs-PDMS are fabricated in this study using the solution casting method with varying concentrations of nano-reinforcements carbon-based SWCNTs to achieve this goal. The solution casting method is used to synthesize SWCNTs-PDMS polymeric membranes for the spectral, thermal, and mechanical efficacy of the resultant. The spectral, thermal stability, and mechanical strength analyses have been performed for enhanced gas permeation at ambient temperature with 20 psi pressure for possible future research on permeability for CO_2_, O_2_, and N_2_ in industrial applications and environmental remediation.

## Materials and methods

### Materials

For the fabrication of gas membranes, toluene, used as a solvent for membrane fabrication, was purchased from SIGMA-Aldrich, UK. Polydimethylsiloxane (PDMS) (Elastosil LR 3003/50 A/B) elastomeric material was purchased from Wacker Chemie Company, Germany. Single-walled carbon nanotubes (SWCNTs) are obtained from United States Research Nanomaterials Inc., USA. All these required chemicals were analytical grade, and purity was 97–99%.

### Fabrication of pure PDMS membrane

**Fig. 1:**
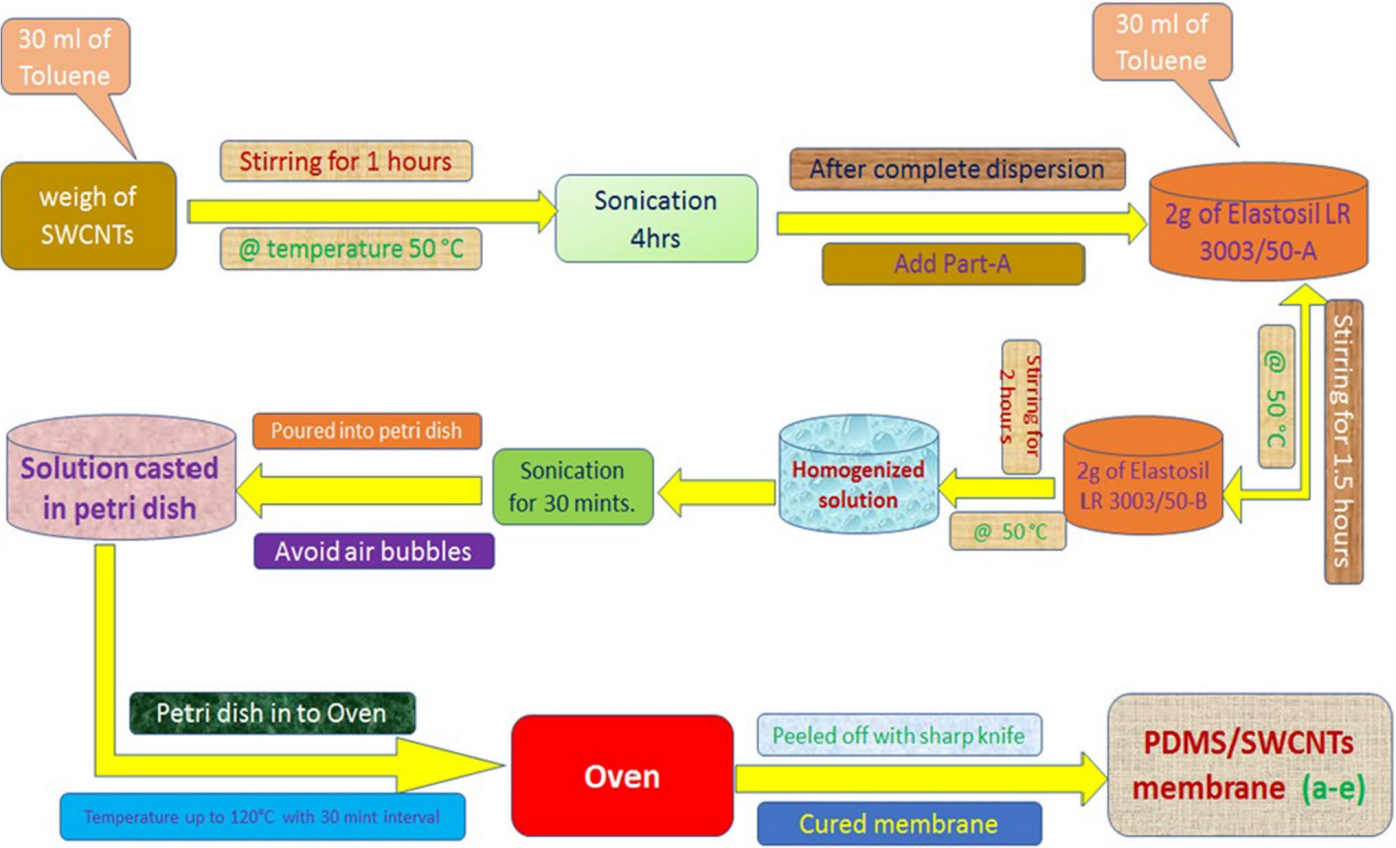
Preparation techniques of PDMS/SWCNTs membrane

A pure PDMS solution was prepared by solution casting method with the addition of 2 g of elastomeric material Elastosil LR 3003/50-A in a toluene solvent of 30 mL at the temperature of 50 °C with constant stirring for 1.5 h. After completely dissolving in the toluene solvent, 2 g of part Elastosil LR 3003/50 B was added at the same temperature of 50 °C for 2 h, stirring the mixture constantly. The prepared viscous solution was then sonicated for 30 min for trapped air removal. The homogenized viscous solution proceeded to fabricate designed polymeric membranes within 4 h. Different ratio % ranges of SWCNTs 0.013, 0.025, 0.038, 0.050, and 0.063 are named A, B, C, D, and E, respectively, fabricated with the same procedure. The viscous solution was slowly poured into the petri dish. Extensive care was taken during pouring to obtain uniform thickness and to avoid air bubbles. The casted membrane petri dish was placed into the oven at 50 °C for 30 min. Then, the temperature was increased to 60 °C for 30 min, after raising the temperature with 10 °C intervals of 30 min up to 120 °C. This process was demonstrated under the controlled evaporation rate, which leads to precipitation. This slow heating process is used to cure the designed membranes without air bubbles trapping the surface of the membrane (Silva et al. 2017). Thus, the resultant PDMS/SWCNTs reinforced membranes were fabricated, and the composite was removed from the petri dish with a sharp knife. The thickness of all prepared membranes was analyzed using a SATRA gauge. These fabricated reinforced nanocomposite membranes have been used to analyze gas permeation performance. Figure 1 presents the overall technique for PDMS/SWCNTs membrane preparation within 4.5 h.

### Characterization

#### Gas permeation process

The fundamental parameters of prepared nanocomposite membranes were used for gas separation membranes, elaborated below.

1. Solubility
2. Permeability
3. Diffusion

The permeation was measured by various gasses. But in this paper, CO_2_, O_2_, and N_2_ were used to analyze the gas permeation.

##### Permeation measurement process

**Fig. 2:**
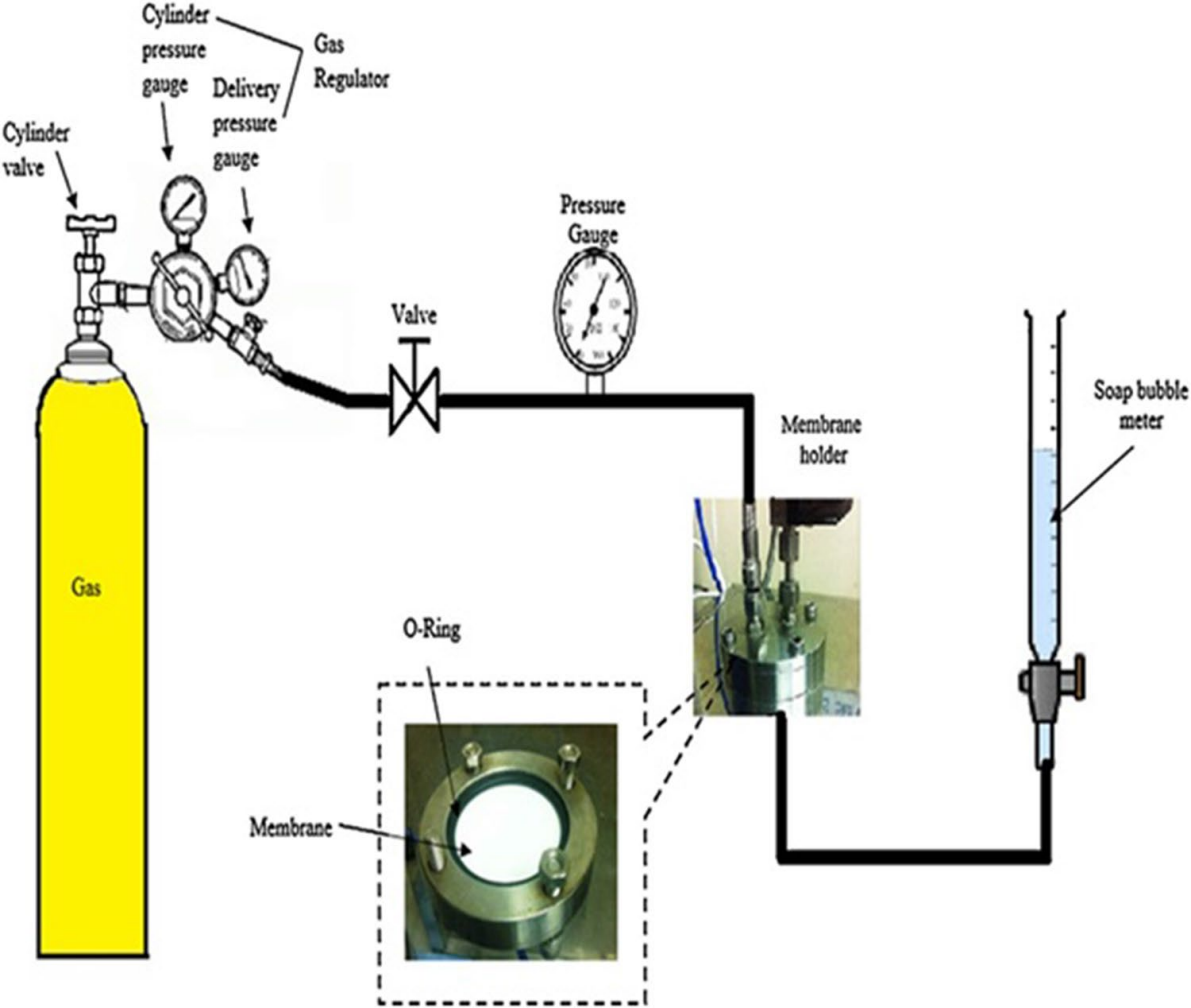
Schematic diagram of gas permeation unit

The gas permeation was measured at room temperature (25 ± 1 °C) with single gas using the constant-volume variable-pressure method in the test chamber (Mahurin et al. 2010). Figure 2 presents the schematic diagram of the gas permeation unit.

The permeation can be measured by below two processes.

1. Variable-pressure and constant-volume technique
2. Variable-volume and constant-pressure technique

Gas permeation tests consist of fabricated membranes mounted on brass disks of 47 mm and have a central hole of 10 mm. The membrane was well-settled and tight in the module. The capillary tube was also adjusted in the module. Then, the flexible pipe was connected with a gas cylinder to measure the permeation. Then, membrane airtightness was done firmly and sealed with epoxy to keep its edges tight. The filter paper was used for mechanical strength, and gas resistance was neglected. All set parameters were checked. Gas was set free from the cylinder by opening the regulator. Then, the required pressure was adjusted to study the permeation. After gas flow, liquid volume in the capillary and time of output permeate was measured to calculate permeation.

The permeation is calculated using Eq. 1. The permeation of other gasses was also measured using the same formula.

1 $$P(Barrer)=\frac{L}{\Delta PA}Q$$

where *Q* = flow rate, *L* = membrane thickness, *A* = area, and *P* = pressure difference.

##### Ideal selectivity

Equation 2 was used to calculate the ideal selectivity *α* of membranes.

2 $${\alpha }_{A/B}=\frac{{P}_{A}}{{P}_{B}}$$

where ideal selectivity is *α*_*A*/*B*_; gas A and gas B permeability is denoted by *P*_*A*_ and *P*_*B*_, respectively.

#### Scanning electron microscopy (SEM)

The SEM was used to analyze the morphology and cross-sectional view of the prepared membranes. The electron beam of required energy was generated from an electron gun and then was focused on the sample by electronegative lenses. The electron beam was scanned on the rectangular-shaped sample. The secondary electrons revealed surface morphology. We got different greyscale images by variation in the number of electrons and electron speed reflected from a different place of the sample (Cazaux 2005). The SEM machine (JSM 6409A, JEOL, Japan) was used, and the sample was gold sputtered.

#### Atomic force microscopy (AFM)

In tapping mode, membrane surface roughness was analyzed using a Multimode AFM (Veeco Metrology Group, Santa Barbara, Canada).

#### Fourier transform infrared spectroscopy (FTIR)

The FTIR was used to analyze the functional groups attached to the polymer or material. The spectrometer in the infrared region can measure the absorbance of transmittance. The qualitative structure of the polymer is studied by wavelength/intensity curves of FTIR (Nowakowski et al. 2008). Fourier transform infrared analysis of membranes, Happ-Genzel instrument detector type DTGS having a resolution of 16, wavenumber at 4000–650 cm^−1^, and background scans are 96 were used.

#### Thermogravimetric analysis (TGA)

TGA was performed to measure the thermal stability of the membrane by using the basic concept of mass loss or decomposition of polymer concerning temperature and time. The sample was exposed to the crucible holder with a microbalance made from platinum, aluminum, quartz, or alumina, which uniformly transferred the heat to the sample. The Perkin Elmer (Diamond 100 TG/DTA, Japan) was used to study the sample. Nitrogen flow was maintained to remove all the corrosive gas at 15 mL·min^−1^, which may take part in the degradation process (Adnan Ahmad et al. 2017, T. Dollase and Spiess 2003).

#### Mechanical strength testing

The mechanical testing was performed to analyze the membrane’s tensile strength, evaluated by CRE-type tester SATRA STM 466, England. The BS EN ISO, 3376–2011 test method followed a 50 mm·min^−1^ extension speed. The maximum force was studied under the controlled environment of 23 ± 2 °C and 45 ± 5% relative humidity. The maximum fractured force was calculated by dividing the cross-sectional area of the sample by its width.

## Results and discussion

### Morphology analysis

**Fig. 3:**
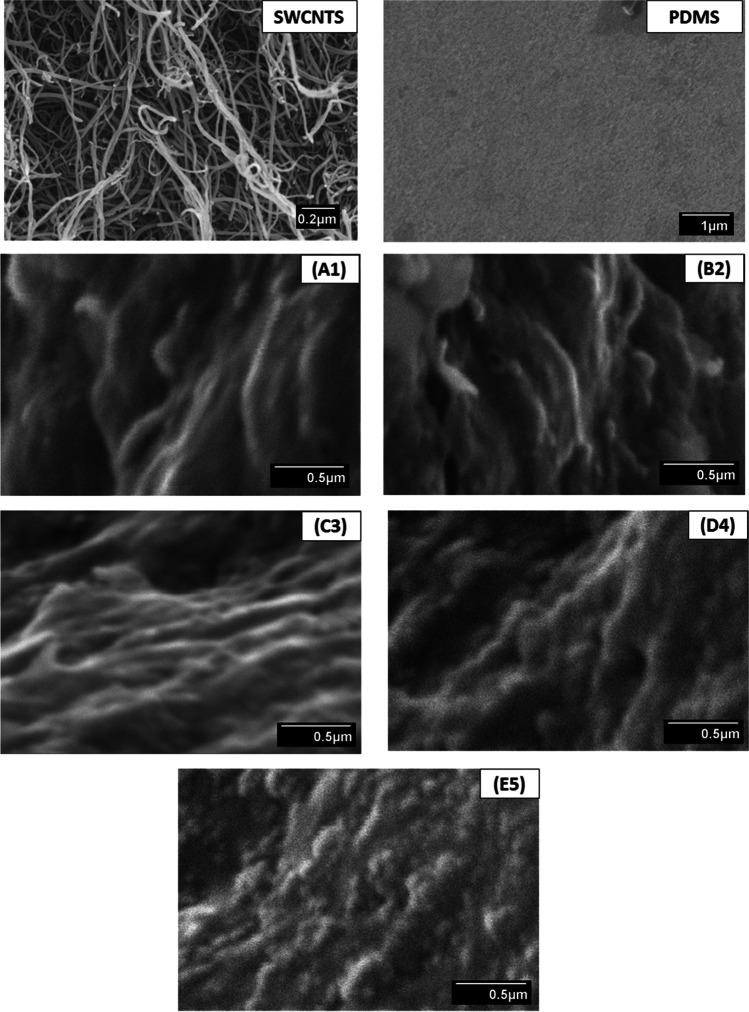
SEM micrographs of PDMS (Pure-N) and SWCNTs (A1, B2, C3, D4, E5) membranes

SEM was used to investigate the efficient permeability of SWCNTs reinforced PDMS membrane (Hajili et al. 2022). The SWCNTs’ surface morphology, uniform dispersion, and porosity are examined to discover the profound reality. The morphological representation of the SEM micrograph is described in Fig. 3 with SWCNTs enhancement regarding concentration. The sample C3, loaded with 0.015 g depicted a smooth surface with no crack, and filler (SWCNTs) was well distributed on the surface compared to other samples. The obtained results also agreed with Afzal et al. (2016) on the effect of the distribution of carbon nanotubes. The D4 sample with SWCNTs concentration showed agglomeration and non-homogeneity of filler particles, as shown in Fig. 3. It demonstrates that the filler is not well instilled in the backbone of PDMS membranes compared to C3, which shows the smooth and even distribution of filler into the membrane structure aligned with the existing literature (Okolo et al. 2020).

### Surface topography

**Fig. 4:**
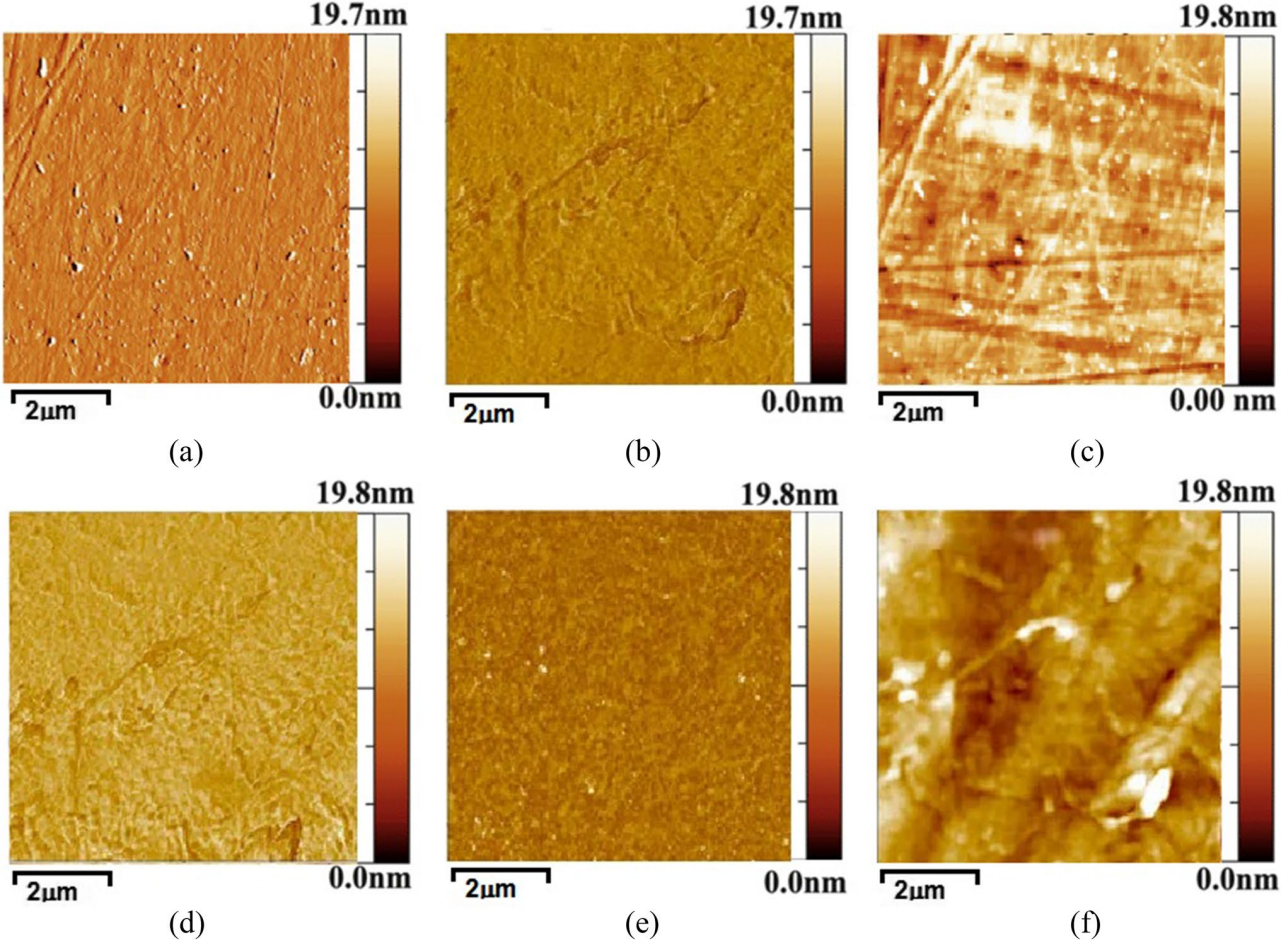
AFM images of surface topography of PDMS-N (**a**) and PDMS/SWCNT-A1 (**b**), PDMS/SWCNT-B2 (**c**), PDMS/SWCNT-C3 (**d**), PDMS/SWCNT-D4 (**e**), PDMS/SWCNT-E5 (**f**) membranes

**Fig. 5:**
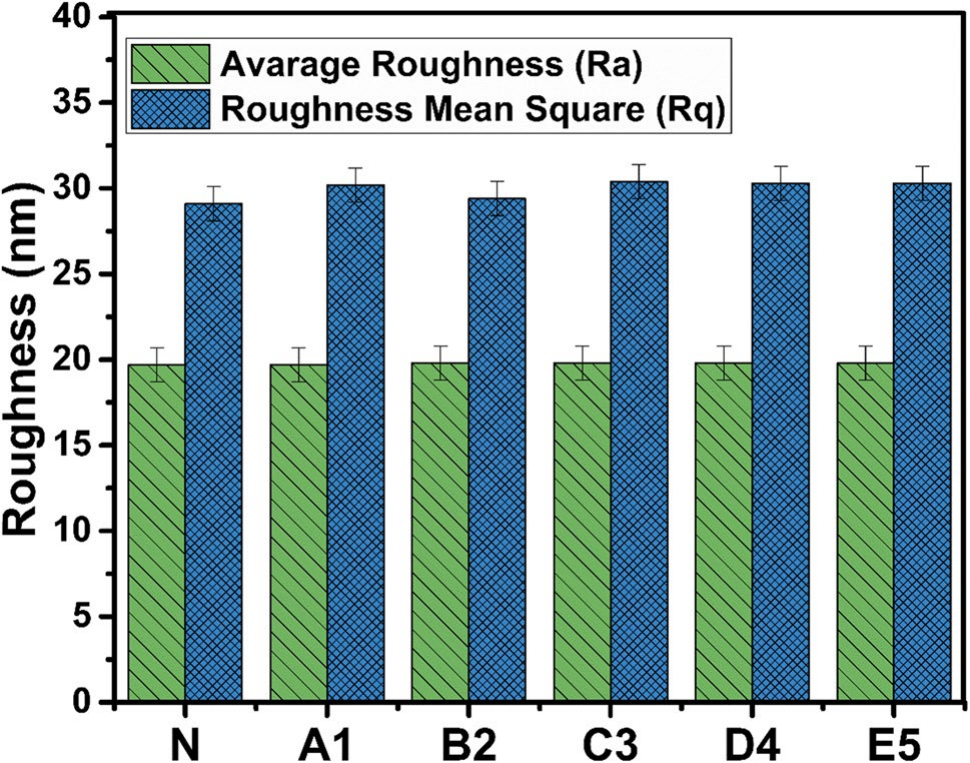
Roughness of membranes’ surface of PDMS-N, PDMS/SWCNT-A1, PDMS/SWCNT-B2, PDMS/SWCNT-C3, PDMS/SWCNT-D4, and PDMS/SWCNT-E5

The atomic force microscopy (AFM) images and surface roughness of the fabricated membranes are shown in Figs. 4 and 5, respectively. Figure 5 presents that the surface roughness on A1, B2, C3, D4, and E5 remains in similar range of ⁓30 nm. Therefore, it is clear from the AFM images and surface roughness that the surface is smooth, and roughness did not fluctuate with the loading of nano-reinforcements. Lower surface roughness indicates higher possibility of gas absorption and lesser fouling on the membrane surface (Iqbal et al. 2018). The results of AFM and roughness analyses are in agreement with SEM micrograph analysis. Therefore, all the nano-reinforced polymeric membranes present gas absorption capacity.

### Structural analysis

**Fig. 6:**
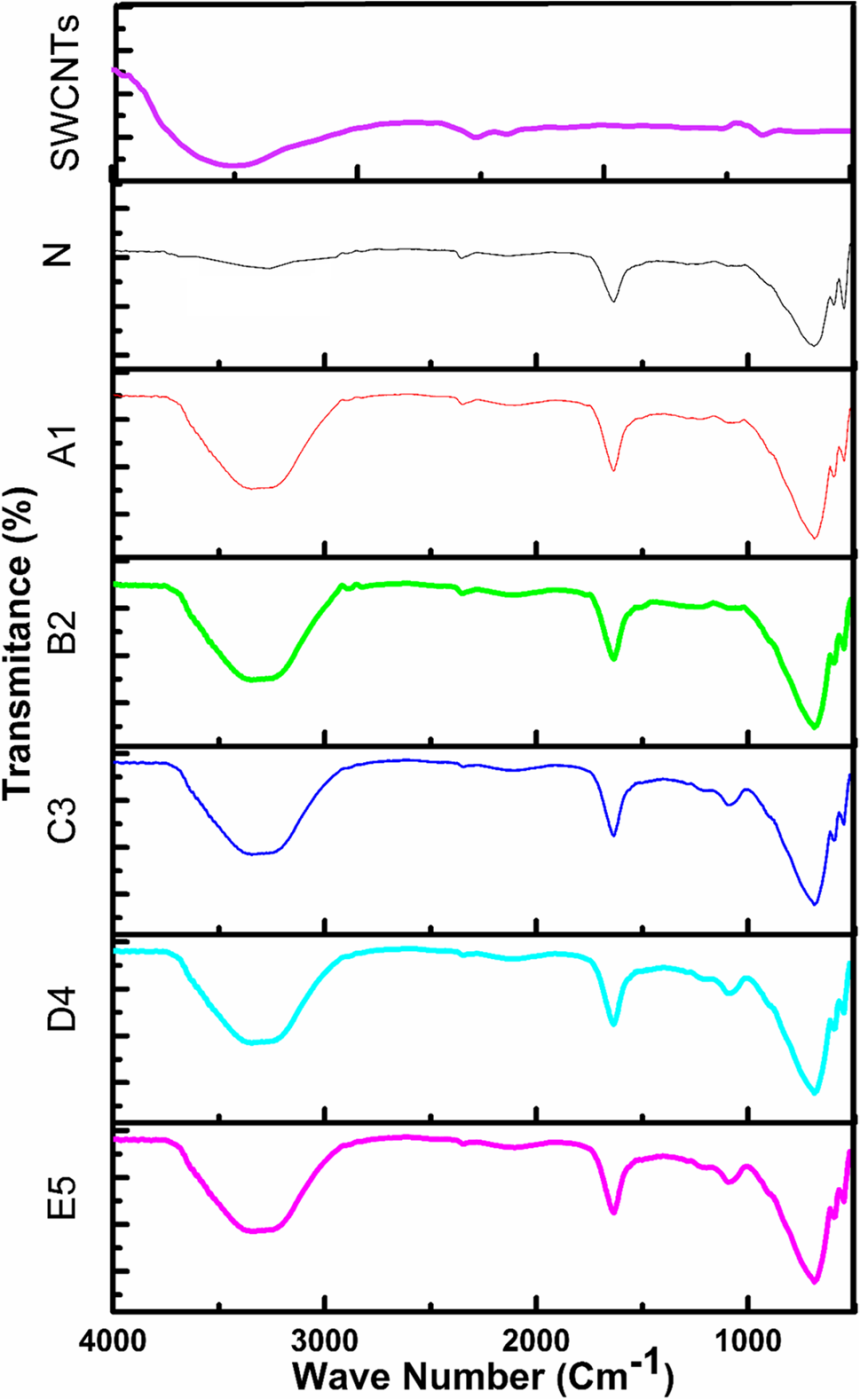
FTIR graph of PURE and SWCNTS-filled membrane

FTIR is employed to investigate the functional group of the fabricated membranes. The IR transmittance spectrum of PDMS-reinforced SWCNTs membrane has been depicted in Fig. 6. Based on the FTIR result, the associated CH_3_ peaks were observed at 1415–1260 cm^−1^. In contrast, the Si–O-Si stretching group was found to range (from 1000 to 1100 cm^−1^), confirmed by the reported literature (Sagar et al. 2015). A lower wavenumber of 930 cm^−1^ appeared with the increased concentration of SWCNTs. This effect can be seen in other materials having carbon. At 835–855 cm^−1^ and 785–815 cm^−1^, Si–C and Si(CH_3_)_2_ peaks have been observed in the FTIR micrograph (Jamshaid et al. 2017).

### Thermal analysis

**Fig. 7:**
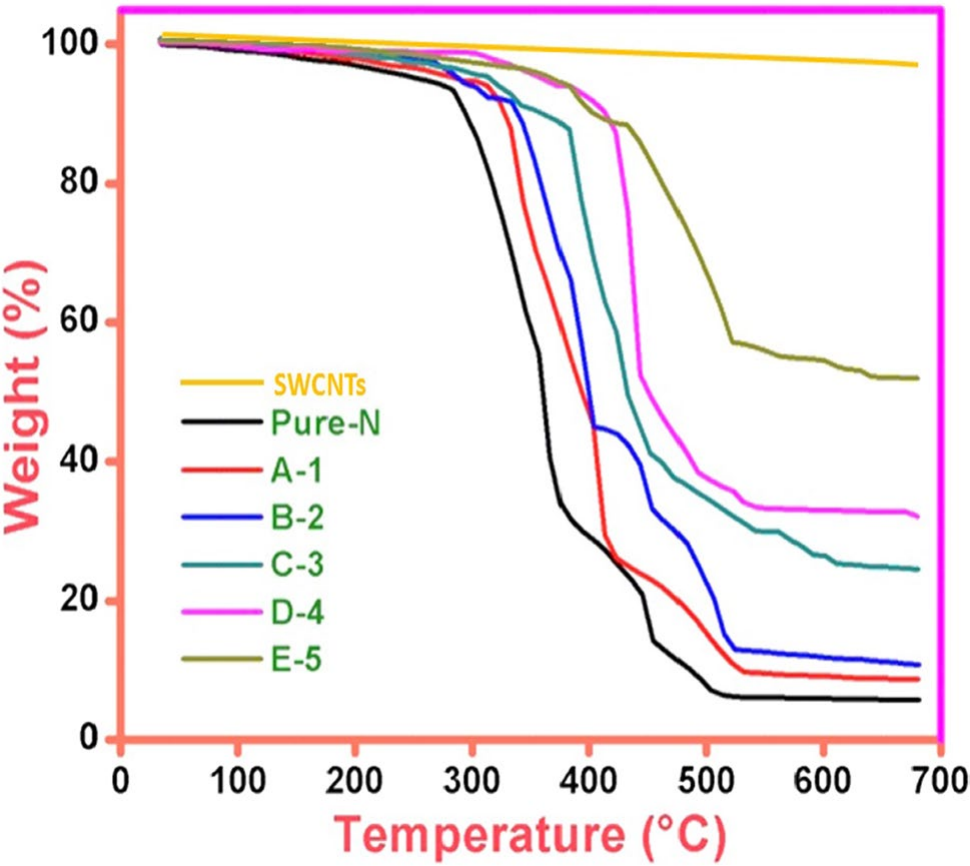
TGA graph of PURE and SWCNTS-filled membrane

TGA study was executed to observe the thermal stability of membranes using Perkin Elmer with the rate of 100 °C·min^−1^. Figure 7 presents the thermal decomposition. The thermogram was achieved by heating in cycles at 300–700 °C with an N_2_ gas flow rate of 15 mL·min^−1^. This gas eliminates all destructive gasses involved in membrane degradation (Iqbal et al. 2018).

Figure 7 depicts that until the 300 °C-onset point, the evaporation of the oxygen-related functional group occurred functionally. Five different compositions of reinforced SWCNTs were fabricated and examined their thermal degradation up to 500 °C. This decomposition took place due to the degradation of the polymer backbone. Based on the thermal profile in Fig. 7, no substantial changes were observed after the offset point at 700 °C. The thermal stability increased as SWCNTs concentration increased in the prepared membrane samples. Still, it showed an adverse effect when it crossed the SWCNTs optimum level, which acted as contamination in the membrane structure (Iqbal et al. 2013). The adverse effect occurred due to contamination depicted in the above TGA graph results. The weight loss % at equilibrium is observed as ⁓45–86% for all fabricated membranes.

### Mechanical strength analysis

**Fig. 8:**
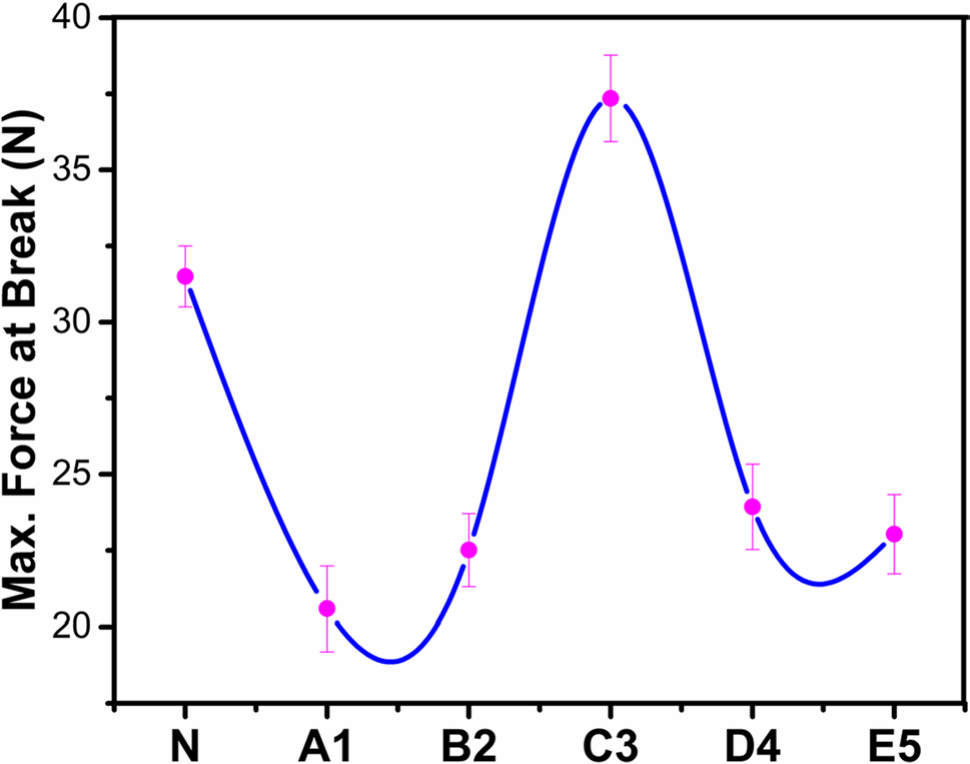
Tensile strength comparison of PURE and SWCNTS-filled membrane

**Fig. 9:**
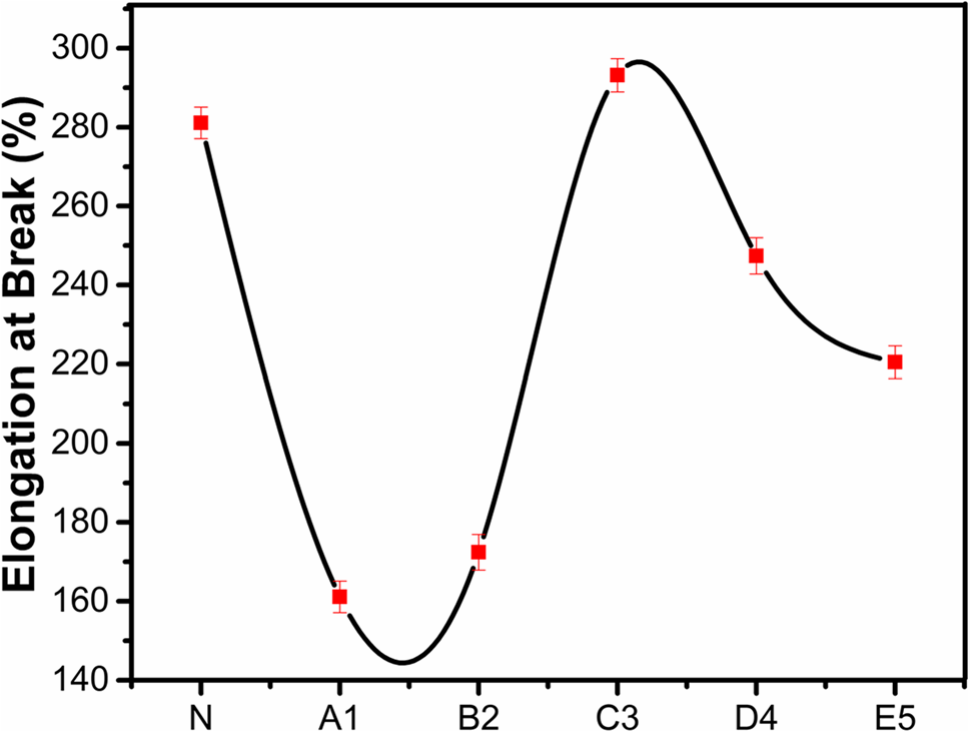
Elongation comparison of PURE and SWCNTS-filled membranes

The SATRA tensile tester STM-566 was used to analyze the breaking strength of the developed membrane. BS EN ISO 3376 test method with jaws separation 50 mm·min^−1^ was used for this purpose. ISO-3376–2011 Modified test method has been used for measuring tensile strength and elongation. CRE-type SATRA STM 566 machine was used for this purpose. Comparison of tensile strength and elongation at break of PURE and SWCNTS-filled membrane were presented in Figs. 8 and 9, respectively.

It was observed that the mechanical features of PDMS membranes were enhanced in the host polymers by increasing the concentration of SWCNTs. But this behavior was not long-lasting as after a specific point, mechanical features get decreased. This behavior was observed due to the excess loading of nanofillers of SWCNTs. This excess was behaving like impurities in the backbone of the PDMS membrane’s structure, which finally tends to the failure of the backbone. This behavior was the same for elongation at break. Hence, sample C3 presented the maximum mechanical characteristics in terms of elongation strength (Berber and Hafez 2016).

### Gas performance evaluation

**Fig. 10:**
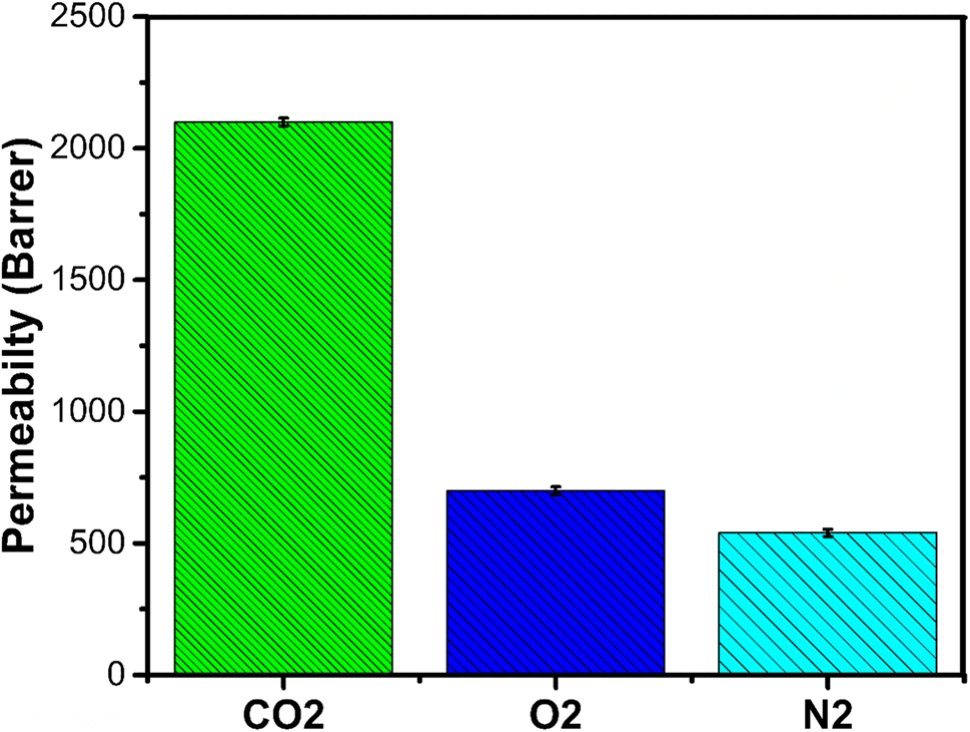
Permeability of gasses with pure PDMS

The PDMS elastomeric material was selected to evaluate gas performance due to its economic cost and good mechanical strength (Robeson 1991). The CNTs, graphene, and zeolite were used to study the gas separation performance (Duval et al.^1994^). Still, we have a lot of room to investigate the peculiar properties of PDMS membranes (Liu and Kulprathipanja 2010). In this study, fabricated PDMS membranes were tested to check gas permeability by high-performance permeability tester CSI-135. The CO_2_, N_2_, and O_2_ gas permeability of the synthesized membrane was measured at ambient temperature (25 °C) at 20 psi pressure. Figure 10 shows the permeability of gasses with pure PDMS.

### Permeation/ideal selectivity results at 25 °C

**Fig. 11:**
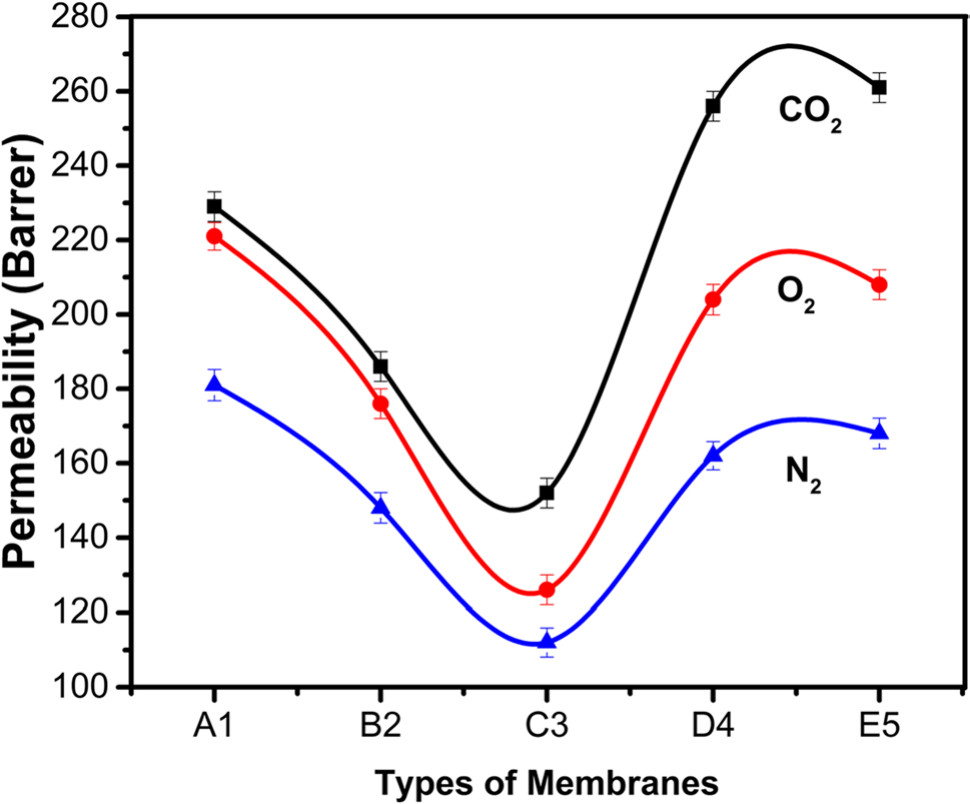
Permeability of gasses with fillers (SWCNTs)

**Fig. 12:**
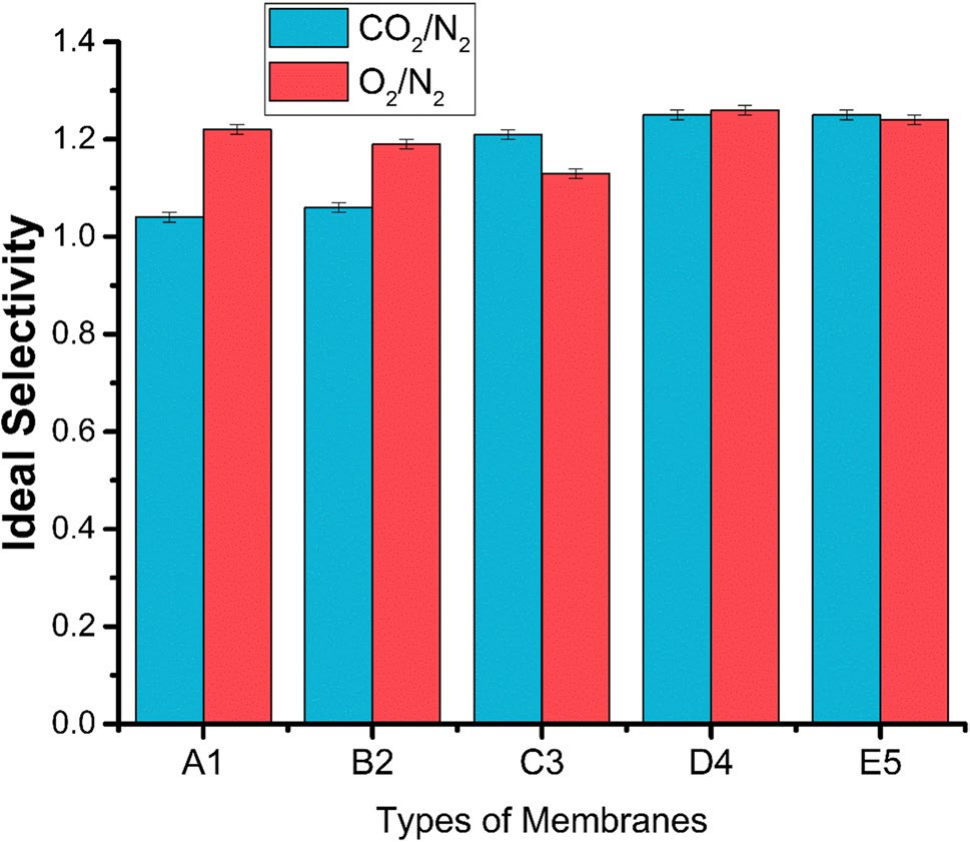
Ideal selectivity of gasses with fillers (SWCNTs)

The gas permeation and ideal selectivity of CO_2_, O_2_, and N_2_ results are denoted in Table 1 at room temperature and graphically represented in Figs. 11 and 12.

**Table 1:** Permeability of gasses with filler (SWCNTs)

Sample #	Permeability (*P*-barrer)			Ideal selectivity	
	CO_2_	O_2_	N_2_	(CO_2_/N_2_)	(O_2_/N_2_)
A-1	229	221	181	1.04	1.22
B-2	186	176	148	1.06	1.19
C-3	152	126	112	1.21	1.13
D-4	256	204	162	1.25	1.26
E-5	261	208	168	1.25	1.24

As depicted in Table 1, Figs. 11 and 12, it has been observed that by increasing nanofillers in PDMS membranes, the permeability of CO_2_, O_2_, and N_2_ decreases, and ideal selectivity gets enhanced (Adrees et al. 2019). The decreasing trend from the first sample was that by increasing the concentration of SWCNTs, the permeability of the B2 and C3 samples was getting down and then abruptly increased. The solution diffusion model interpreted in terms of solubility and diffusivity of gas molecules in individual polymers explains membrane permeability and ideal selectivity. The graphical representation shows the inverse relationship of gas permeability and SWCNTs as the increasing concentration of SWCNTs permeability decreases. Permeability was reduced because SWCNTs nanofiller have high chain compactness, low segmental motion, and small free volume. It tends to decrease chain mobility and diffusivity of gas molecules in nanofiller-incorporated membranes due to these properties of SWCNTs. The observation declined permeability by enhancing the content of SWCNTs in synthesized membranes.

## Conclusions

PDMS/SWCNTs polymeric composite membranes were fabricated using the thermally induced phase separation process, varying the SWCNTs nano-reinforced filler concentration ratio. The resultant membranes had excellent thermal stability with optimized mechanical for gas selectivity and permeation performance at ambient temperature with 20 psi pressure for CO_2_, O_2_, and N_2_, respectively. The 0.063 wt.% SWCNTs membrane (E5) was manifested as the best performing membrane for CO_2_ gas permeability and CO_2_/N_2_ idea selectivity, 0.013 wt.% SWCNTs membrane (A1) was the best for O_2_ and N_2_ permeability and 0.050 wt.% SWCNTs membrane (D4) mixed gas O_2_/N_2_ selectivity. By increasing the concentration of the nanofiller of SWCNTs in PDMS, the permeability tends to decrease, and then after a particular value of nanofiller incorporation, permeability gets enhanced. The increase of pore size owing to the higher concentration of nanofiller in host polymeric materials may act as an impurity that led to pore generation at a pressure, which is the reason behind the above behavior. Selectivity gave the same outcomes through the fabricated membranes. Thermal stability is enhanced with the incorporation of SWCNTs nanofiller.

On the other hand, the mechanical characteristics follow the same trend as permeability due to the nanofiller’s overloading. The 0.038 wt.% reinforced SWCNTs give the maximum tensile force, 37.350 N, tensile strength, 8.893 N·mm^−2^, and elongation at break, 293.149 mm, while 0.013 wt.% has low tensile force, strength, and elongation at break. Therefore, the outcomes of this designed novel research revealed that SWCNT/PDMS polymeric nanocomposite membranes are compatible and hence facilitate the development of a homogeneous dense film structure with excellent potential for efficient membranes suitable for targeted gas separation with high commercial and environmental applications.

## Data Availability

The data that support the findings of this study are available from the corresponding author, Nazia Hossain, upon reasonable request.

## Abbreviations

PDMS: Polydimethylsiloxane
SWCNTs: Single-walled carbon nanotubes
TGA: Thermogravimetric analysis
CO_2_: Carbon dioxide gas
O_2_: Oxygen gas
N_2_: Nitrogen gas
CH_4_: Methane
SEM: Scanning electron microscopy
AFM: Atomic force microscopy
FTIR: Fourier transform infrared analysis
MST: Mechanical stability testing
